# Reply to Jensen, O.K. On the Use of Quantitative Sensory Testing to Estimate Central Sensitization in Humans. Comment on “Schuttert et al. The Definition, Assessment, and Prevalence of (Human Assumed) Central Sensitisation in Patients with Chronic Low Back Pain: A Systematic Review. *J. Clin. Med.* 2021, *10*, 5931”

**DOI:** 10.3390/jcm11082113

**Published:** 2022-04-11

**Authors:** Ingrid Schuttert, Hans Timmerman, Kristian K. Petersen, Megan E. McPhee, Lars Arendt-Nielsen, Michiel F. Reneman, André P. Wolff

**Affiliations:** 1 Pain Center, Department of Anaesthesiology, University Medical Center Groningen, University of Groningen, 9750 RA Groningen, The Netherlands; i.schuttert@umcg.nl (I.S.); h.timmerman02@umcg.nl (H.T.); 2 Center for Neuroplasticity and Pain, Department of Health Science and Technology, Faculty of Medicine, Aalborg University, DK-9220 Aalborg, Denmark; kkp@hst.aau.dk (K.K.P.); mmp@hst.aau.dk (M.E.M.); lan@hst.aau.dk (L.A.-N.); 3 Department of Medical Gastroenterology (Mech-Sense), Aalborg University Hospital, DK-9220 Aalborg, Denmark; 4 Department of Rehabilitation Medicine, University Medical Center Groningen, University of Groningen, 9750 RA Groningen, The Netherlands; m.f.reneman@umcg.nl

We thank Dr. Jensen for his interest [1] in our systematic review [2]. Dr. Jensen states that we introduce a new phenomenon called human-assumed central sensitization (HACS), though we merely wanted to introduce a new term that, in our opinion, better reflects the current state of science.

Dr. Jensen is correct that no articles on digital tender point (TP) examination were included. No data were found after re-examining the results of the initial search regarding the references given in Dr. Jensen’s comment [2]. Moreover, considering the studies suggested by Dr. Jensen, we feel that they were correctly not included in our systematic review [2] based on the inclusion criteria. One study included patients with fibromyalgia [3], but not patients with chronic low back pain. Four studies were not based on (the assessment of) HACS [4,5,6,7]. Finally, one study [8] mentioned HACS in the discussion to explain the results of the study, but did not assess HACS.

The suggested cut-off points for TP examination [8] are for the women and men separately, which is necessary to account for sex differences that are present in pain [9,10,11,12]. TP may be associated with fibromyalgia [13,14,15], but the association with HACS has not been established. This suggested connection seems to be made based on the altered pain processing, which could also be a result of nociplastic pain [16], the third mechanistic descriptor for chronic pain states. The terms HACS and nociplastic pain overlap but are not synonymous [17]. Furthermore, there are no gold standards to demonstrate the presence of nociplastic pain and HACS in patients with chronic pain.

TP examination could be interesting as an assessment for HACS, but a clear distinction should be made about TP examination being used to identify fibromyalgia or widespread pain and the assessment of HACS. Adding TP examination to the list of indicator tests could probably provide more insight into the presence of HACS. However, more research is needed in patients with chronic low back pain where TP examination is used to assess the underlying pain mechanisms of HACS in combination with other indicator tests, such as the central sensitization inventory and other quantitative sensory testing instruments.

## Data Availability

The authors of the original papers can request all data and related metadata underlying this review’s findings. Data can also be requested from the first author of this systematic review, but data will only be provided with the original data authors consent.
